# Supplementary material for: Identification of photoperiod-induced specific miRNAs in the adrenal glands of Sunite sheep (Ovis aries)
Source: Front Vet Sci. 2022 Jul 22;9:888207. doi: 10.3389/fvets.2022.888207 (PMC9354845; doi:10.3389/fvets.2022.888207)
Supplement: Supplementary file 1 [file Data_Sheet_1.ZIP › Supplementary file/miRDeep2 file.zip!/miRDeep2 file/Know_Structure/SP42A3/oar-mir-376c.pdf]

miRBase precursor : oar-mir-376c
Total read count : 405
oar-miR-376c-5p read count : 144
oar-miR-376c-3p read count : 261
remaining reads : 0

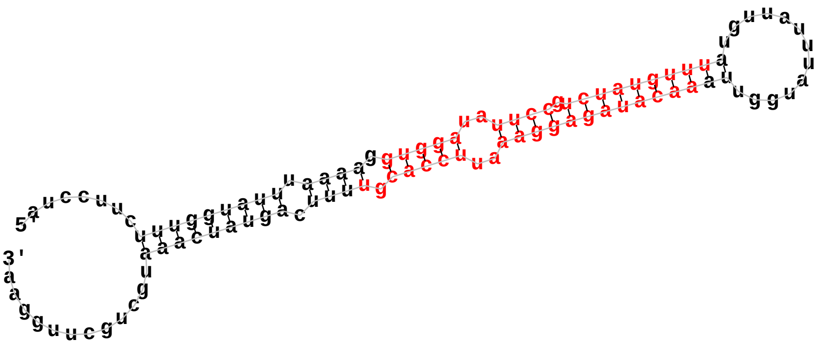

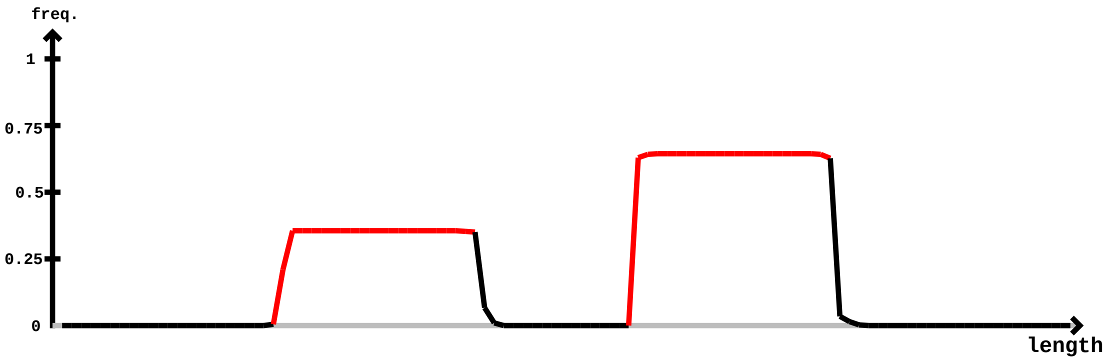


```
                                                                oar-miR-376c-3p
                         oar-miR-376c-5p
5'- auccuucuuugguauuuaaaagguggauauuccgucuauguuuauguuauuuaugguuaaacauagaggaaauuccacguuuucaguaucaaaugcugcuuggaa -3'  exp
    .......(((((((((.((((.(((((..((((.((((((((((............))))))))))))))..))))).)))).)))))))))............. reads  mm  sample
    .....................gguggauauuccgucuauguuu.............................................................. 2      0   seq
    ......................guggauauuccgucuaugu................................................................ 1      0   seq
    ......................guggauauuccgucuauguuu.............................................................. 69     0   seq
    ......................guggauauuccgucuauguuua............................................................. 12     0   seq
    ......................guggauauuccgucuauguuuau............................................................ 1      0   seq
    .......................uggauauuccgucuauguu............................................................... 1      0   seq
    .......................uggauauuccgucuauguuu.............................................................. 44     0   seq
    .......................uggauauuccgucuauguuua............................................................. 11     0   seq
    .......................uggauauuccgucuauguuuau............................................................ 3      0   seq
    ...........................................................aacauagaggaaauuccac........................... 1      0   seq
    ...........................................................aacauagaggaaauuccacg.......................... 6      0   seq
    ...........................................................aacauagaggaaauuccacgu......................... 240    0   seq
    ...........................................................aacauagaggaaauuccacguu........................ 6      0   seq
    ...........................................................aacauagaggaaauuccacguuu....................... 2      0   seq
    ............................................................acauagaggaaauuccacguu........................ 2      0   seq
    ............................................................acauagaggaaauuccacguuu....................... 3      0   seq
    .............................................................cauagaggaaauuccacguuuu...................... 1      0   seq
```
